# Early-Onset Sepsis in Neonates - A Population-Based Study in South-West Norway From 1996 to 2018

**DOI:** 10.3389/fped.2021.634798

**Published:** 2021-03-17

**Authors:** Anlaug Vatne, Claus Klingenberg, Siren Rettedal, Knut Øymar

**Affiliations:** ^1^Department of Pediatrics, Stavanger University Hospital, Stavanger, Norway; ^2^Department of Clinical Science, University of Bergen, Bergen, Norway; ^3^Department of Paediatrics, University Hospital of North Norway, Tromsø, Norway; ^4^Paediatric Research Group, Department of Clinical Medicine, University of Tromsø-The Arctic University of Norway, Tromsø, Norway

**Keywords:** infection, early-onset sepsis, neonatal sepsis, antibiotic therapy, antibiotic resistance, antibiotic susceptibility

## Abstract

**Background:** The epidemiology of early-onset sepsis (EOS) may change over time. Longitudinal surveillance of causative pathogens, antibiotic susceptibility patterns and antibiotic therapy is important for optimal therapy strategies.

**Objectives:** To describe the incidence of culture-confirmed EOS, causative pathogens, antibiotic susceptibility patterns and antibiotic therapy over a 23-year period.

**Methods:** Retrospective population-based study from a single-center neonatal intensive care unit at Stavanger University Hospital, Norway, covering a population in South-West Norway, during the 23-year period 1996–2018.

**Results:** Of 104,377 live born infants, 101 infants (0.97/1,000) had culture-confirmed EOS; 89 with Gram positive and 12 with Gram-negative bacteria. The EOS-attributable mortality was 6/101 (5.8%). For the three most prevalent pathogens the incidences were; Group B streptococcus (GBS) 0.57/1,000, *Escherichia coli* 0.11/1,000 and viridans group streptococci (VGS) 0.10/1,000. GBS was the most common pathogen (59/93; 63%) in infants with gestational age (GA) ≥ 28 weeks. In contrast, among extremely preterm infants (GA <28 weeks) the incidence of *E. coli* infection was higher than for GBS infection. The second most common bacterial pathogens causing EOS among term infants were VGS. There was no change in the incidence of EOS for the entire study period, but from 2000 to 2018 there was a mean decline in EOS by 6% per year (95% CI 1%−10%) (*p* = 0.019). The incidences of GBS and *E. coli* did not change during the study period. The initial empirical antibiotic regimen for EOS was in all cases a combination of benzylpenicillin or ampicillin and an aminoglycoside, but in 21/101 (21%) of cases a broad-spectrum antibiotic was either added or substituted this regimen. In 2/101 (2%) EOS cases, the pathogens were nonsusceptible to the empirical antibiotic regimen. All *E. coli* isolates were susceptible to aminoglycosides.

**Conclusion:** GBS was the most common causative pathogens in EOS, but *E. coli* dominated in infants with GA <28 weeks. There was no change in the incidence of EOS during the entire study period. The current empiric regimen with benzylpenicillin and gentamicin provides a very high coverage for EOS in our setting.

## Introduction

Early-onset sepsis (EOS) remains a major contributor to neonatal morbidity and mortality (1). Although most EOS cases occur in term infants, incidence and infection-attributable mortality is higher in preterm infants, inversely related to gestational age (GA) (2). In many countries and regions the incidence of EOS has decreased in the past decades, in particular after implementing effective intrapartum antibiotic prophylaxis (1, 3–6). Among term (GA ≥ 37 weeks) and moderately preterm infants (GA 28–36 weeks) with EOS, group B streptococci (GBS) are the dominant pathogens identified in blood cultures (5–7). In contrast, among extremely preterm infants (GA <28 weeks), *Escherichia coli* is often the most frequently detected pathogen (5). Patterns of other bacterial pathogens causing EOS are less well-described.

In infants with suspected EOS, empirical antibiotic therapy is commenced before blood culture results are available. Longitudinal surveillance for identification of changes in causative pathogens, clinical outcomes including mortality, and antibiotic susceptibility is important when tailoring optimal prevention and empiric therapy strategies (5, 8, 9). However, data on antibiotic susceptibility is often not reported in epidemiological studies. There is growing concern about increasing antibiotic nonsusceptibility among pathogens causing EOS, especially in Gram-negative pathogens where often few therapeutic options are available (10). Increasing nonsusceptibility rates, could potentially threaten the effectiveness of standard empiric regimens (5, 10–12). In the action plan to combat antibiotic resistance, the World Health Organization calls for increased knowledge on local epidemiology and antibiotic susceptibility patterns (9).

In this population-based study including more than 100,000 live born (LB) infants, we aimed to describe the incidence of culture-confirmed EOS, causative pathogens, antibiotic susceptibility patterns and antibiotic therapy over 23 years in South-West Norway.

## Materials and Methods

### Setting

Stavanger University Hospital in South-West Norway is the only hospital for a well-defined population of around 370,000 inhabitants, offering primary, secondary and tertiary obstetric and neonatal intensive care. There have been 4,000–5,000 annual deliveries during the last decades. All infants born in the catchment area and who receive intravenous antibiotic therapy for EOS have been admitted to the neonatal intensive care unit (NICU) in Stavanger.

### Study Design, Participants, and Data Collection

This is a single-center, population-based retrospective study over a 23-year period from January 1996 to December 2018. The annual number of live births with a GA of ≥22 weeks during the study period were obtained from the maternity unit. Newborn infants with positive blood cultures obtained during the first 72 h of life were identified by the local microbiology laboratory blood culture registry, and causative pathogens and antibiotic susceptibility were registered. Detailed clinical information was extracted from the medical records for all infants with culture-confirmed EOS, including GA, birthweight (BW), symptoms and signs of EOS, infection-attributable mortality, maximum value of C-reactive protein (CRP), choice of antibiotics and duration of therapy. The diagnosis of clinical chorioamnionitis was extracted from the mother's medical record.

### Definitions, Microbiology, and Antibiotic Therapy

EOS was defined as growth of pathogenic bacteria in a blood culture obtained ≤ 72 h of life, and treatment with antibiotics ≥5 or <5 days if death occurred (1). Culture-negative EOS is a controversial diagnosis (13), and we did not include cases coined as culture-negative sepsis also due to lack of a uniform consensus definition. EOS-attributable mortality was defined as death occurring within 7 days after growth of pathogenic bacteria in blood culture where sepsis was the assumed cause. The incidence of EOS was defined as cases of EOS per 1,000 LB infants. Clinical chorioamnionitis was prospectively diagnosed by the responsible obstetrician, and for this study the clinical diagnosis was obtained from the medical records. Cases later diagnosed as histological chorioamnionitis were not included. Infants were classified as symptomatic if they had signs of EOS, and the time from birth to onset of symptoms was registered.

Blood cultures were obtained prior to initiation of antibiotic therapy using BacT/ALERT PF Plus Aerobic Pediatric culture bottles (BioMérieux, Inc., Durham, NC) throughout the study period. Matrix-assisted laser desorption ionization - time-of-flight (MALDI-TOF) mass spectrometry was introduced in 2012, gradually replacing traditional phenotypic species identification. Micrococci, propionibacteria, corynebacteria, or diphtheroids grown alone in a single culture, growth of more than one bacteria, and all coagulase-negative staphylococci (CoNS) were considered contaminants (1). Pathogens were grouped into Gram-positive and Gram-negative bacteria. Viridans group streptococci (VGS) include *Streptococcus mitis* and *Streptococcus alactolyticus* and “Other streptococci” in this report include *Streptococcus pyogenes* and *Streptococcus pneumoniae*. A blood culture pathogen was defined as susceptible to an antibiotic when the final interpretation report indicated S (susceptible) and nonsusceptible when the report indicated R (resistant) or I (intermediate). Antibiotic susceptibility testing followed the guidelines from the Norwegian working group for antibiotics (14), closely aligned with the EUCAST criteria (15).

The local empirical antibiotic regimen for EOS consisted of ampicillin in combination with an aminoglycoside (tobramycin or gentamicin) from 1996 to 2010, and benzylpenicillin and gentamicin from 2011 to 2018.

### Ethical Considerations

The local institutional review board and data protection officer approved the study as a local quality improvement project that did not need approval by the regional ethics committee.

### Statistical Analyses

Data were analyzed using IBM-SPSS version 24 statistical software (IBM, Armonk NY, USA). Results are expressed as mean with 95% confidence interval (CI) or median with interquartile range (IQR), as appropriate. Differences between groups and time periods (1996–2006 vs. 2007–2018) were analyzed with *t*-test or Mann-Whitney test as appropriate for continuous data, and the chi-square test or Fisher-exact test for categorical data. Regression models were used to test for trends over time (linear) where year was the continuous predictor. All tests were two-tailed. *P*-values of <0.05 were considered statistically significant.

## Results

### Incidence and Causative Pathogens

**Figure 1:**
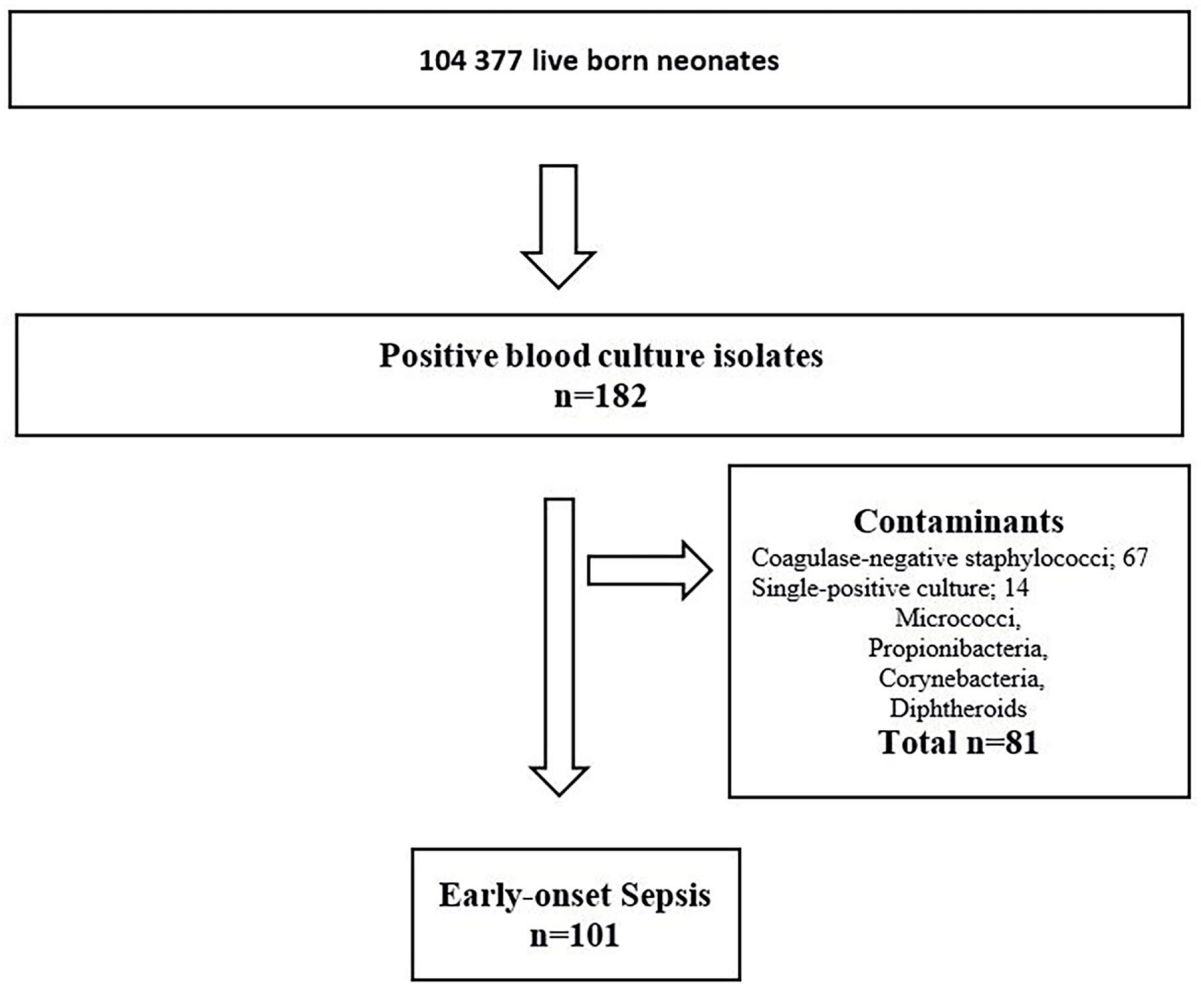
Flowchart – Infants diagnosed with early-onset sepsis in a population-based observational study at Stavanger University Hospital, Norway, 1996–2018.

**Figure 2:**
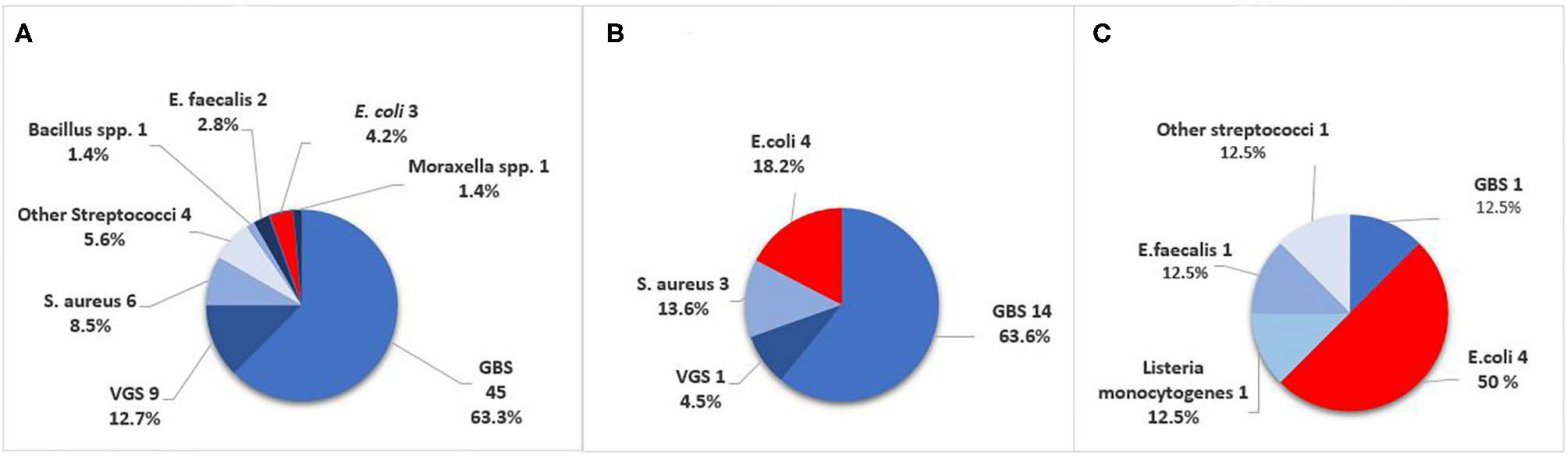
Causative pathogens in culture-confirmed early-onset sepsis (EOS) in infants born at Stavanger University Hospital, 1996–2018. **(A)** GA ≥ 37 weeks. **(B)** GA 28–36 weeks. **(C)** GA <28 weeks. GBS, Group B Streptococci; VGS, viridans group streptococci.

During the 23-year study period, 104,377 infants were LB. Of these; 96,024 (92%) infants were born at term, and 8,353 (8%) infants were born preterm before 37 weeks gestation. Among the preterm infants, 7,890 (7.6%) had GA between 28–36 weeks and 463 (0.4%) GA <28 weeks. There were 101 infants with culture-confirmed EOS (Figure 1). The overall incidence of EOS, incidence by causative pathogens and incidence for different groups of GA are presented in Table 1 and with subgroups in Figure 2. Most cases of EOS were among term infants; 71/101 (70%), but the incidence was higher among preterm infants (Table 1). Compared to term infants, the incidence of EOS in moderately preterm (GA 28–36 weeks) and extremely preterm (GA <28 weeks) infants were 3.9 and 24-fold higher, respectively.

**Table 1:** Incidence of early-onset sepsis (EOS) per 1000 live births among infants born at Stavanger University Hospital during 1996–2018.

	**All EOS isolates**	**GBS**	***Escherichia coli***	**VGS**
**Infants**	**Number infants incidence (95% CI)**	**Number infants incidence (95% CI**	**Number infants incidence (95% CI**	**Number infants incidence (95% CI**
All	*N* = 101	*N* = 60	*N* = 11	*N* = 10
*N* = 104,377	0.97 (0.71, 1.23)	0.57 (0.39, 0.75)	0.11 (0.03, 0.18)	0.10 (0.06, 0.19)
GA ≥ 37 weeks	*N* = 71	*N* = 45	*N* = 3	*N* = 9
*N* = 96,024	0.74 (0.52, 0.96)	0.47 (0.31, 0.63)	0.03 (0.02, 0.09)	0.084 (0.014, 0.18)
GA 28–36 weeks	*N* = 22	*N* = 14	*N* = 4	*N* = 1
*N* = 7,890	2.8 (1.43, 4.28)	1.8 (0.66, 3.0)	0.51 (0.01, 0.92)	0.13
GA <28 weeks	*N* = 8	*N* = 1	*N* = 4	*N* = 0
*N* = 463	17.8 (6.2, 29.4)	2.2	8.2 (0.13, 16.3)	

*Incidence; case per 1,000 live births*.

*GA, Gestational age, median (IQR); GBS, group B streptococci; VGS, viridans group streptococci*.

Among term and moderately preterm infants, GBS was a more frequent cause of EOS than *E. coli*. In extremely preterm infants however, there were four cases of *E. coli* infection and only one GBS case (Table 1). The incidence of Gram-negative pathogens decreased by increasing weeks of GA (OR 0.79, 95% CI 0.69–0.89, *p* < 0.001). VGS was the second largest group of pathogens with 10/101 cases (10%), predominantly occurring in term infants (9/10).

**Figure 3:**
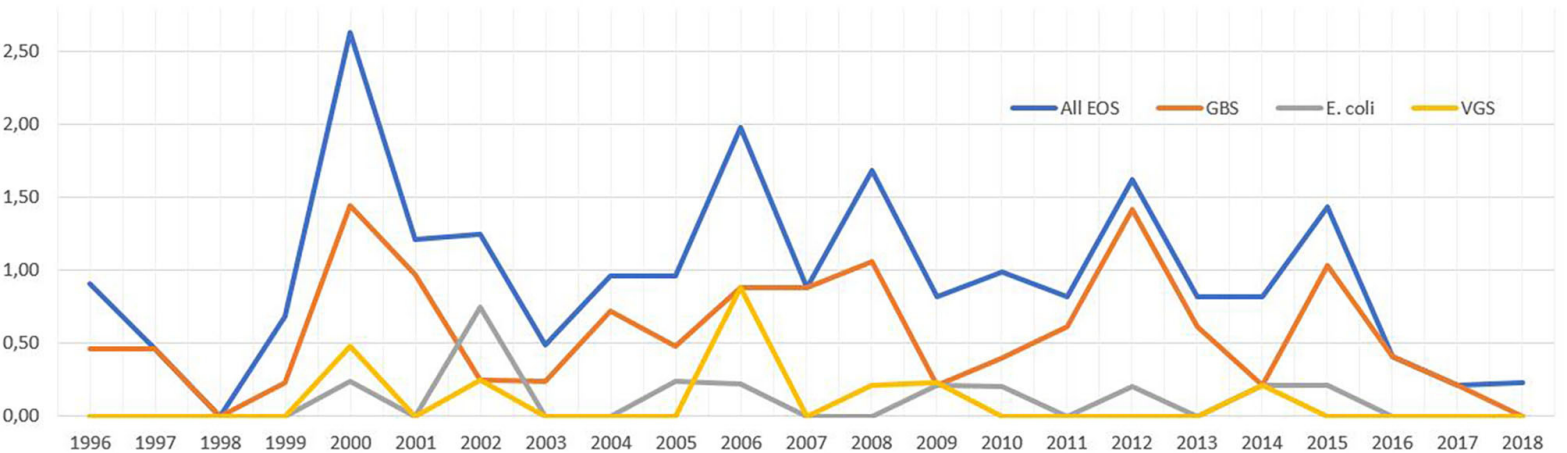
Annual incidence of early-onset sepsis (EOS) per 1,000 live born caused by all pathogens, Group B streptococci (GBS), *Escherichia coli* and viridans group streptococci (VGS) during 1996-2018. GBS, Group B Streptococci; VGS, viridans group streptococci.

The yearly incidence of EOS for different pathogens are shown in Figure 3. There was no difference in the total incidence of EOS between the two periods 1996–2006 and 2007–2018 (1.05/1,000 vs. 0.90/1,000, *p* = 0.49). There was no change in the incidence during the study period for all infants with EOS or for EOS caused by different pathogens (data not shown). However, for the period from 2000 through 2018 analyzed separately, there was a mean decline in the incidence of EOS by 6% per year (95% CI 1–10%) (*p* = 0.019). There was no change in the incidence of EOS during the study period for any of the GA groups when analyzed separately (data not shown).

### Clinical Characteristics and Mortality

In total, 14/101 infants with EOS had been exposed to chorioamnionitis; 10/71 (14%) term, no moderately preterm, and 4/8 (50%) extremely preterm infants. Among infants with EOS exposed to chorioamnionitis, all extremely preterm infants and 7/10 term infants developed symptoms of EOS within the first 6 h of life. Overall, 95/101 (94%) infants with EOS had onset of symptoms within the first 24 h of life. The median (IQR) time to start of EOS-symptoms was 3.0 h (1.0, 13.0). The proportion of infants with symptoms at birth was higher among preterm 18/30 (60%) compared to term infants 26/71 (37%), *p* = 0.047.

The mortality and maximum value of CRP for different groups of GA with EOS are shown in Table 2. Six infants with EOS died; five of these were born preterm. The median (IQR) GA in infants who died was 34 (26–36) weeks. The EOS- attributable mortality was higher in infants with GA <28 weeks compared to term infants (*p* = 0.025). The maximum CRP values were higher in infants with Gram-positive compared to Gram-negative EOS, *p* = 0.003.

**Table 2:** Early-onset sepsis (EOS) attributable mortality and maximum C-reactive protein (CRP) levels among infants of different gestational age (GA) born at Stavanger University Hospital, 1996–2018.

	**All cases EOS**		**Gram-positive EOS**		**Gram-negative EOS**	
	**Deaths/Cases (%)**	CRP^*^ (mg/L)	**Deaths/Cases (%)**	CRP^*^ (mg/L)	**Deaths/Cases (%)**	CRP^*^ (mg/L)
All infants	6/101 (5.8%)	65 (39–110)	3/89 (3.3%)	67 (44–120)	3/12 (25%)	30 (11–62)
GA ≥ 37 weeks	1/71 (1.4%)	65 (43–122)	1/67 (1.5%)	67 (47–126)	0/4 (0%)	22 (5–39)
GA 28–36 weeks	3/22 (13%)	71 (22–99)	2/18 (11%)	65 (10–96)	1/4 (25%)	55 (17–114)
GA <28 weeks	2/8 (25%)	45 (13–112)	0/4 (0)	92 (20–167)	2/4 (50%)	30 (10–60)

*IQR, interquartile range; EOS, Early-onset sepsis; GA, gestational age*.

* *C-reactive protein (CRP) values are the highest values reported during the sepsis episode and presented as median and interquartile range (mg/L)*.

### Antibiotic Therapy and Susceptibility

The antibiotic regimen for EOS was in all cases a combination of benzylpenicillin or ampicillin and an aminoglycoside. In 21/101 (21%) of cases, a broad-spectrum antibiotic was either added or substituted later the empiric regimen. There was no change in the number of infants given broad-spectrum antibiotics between the periods 1996–2006 and 2007–2018.

The median (IQR) duration of antibiotic therapy for EOS declined from 14 (10–14) days in the period 1996–2006 compared to 8 (7–10) days in the period 2007–2018 (*p* < 0.013).

All GBS isolates were susceptible to benzylpenicillin, and all *E. coli* isolates were susceptible to both gentamicin and a third-generation cephalosporin. No Gram-negative isolates produced extended-spectrum beta-lactamase (ESBL). 10/11 (91%) of *E. coli* isolates were nonsusceptible to ampicillin (Table 3).

**Table 3:** Antibiotic nonsusceptibility rates in cases with early-onset sepsis at Stavanger University Hospital, 1996–2018.

	**Nonsusceptible**
***Escherichia coli*** **(*****n*** **=** **11)**	
• Ampicillin	10/11 (91%)
• Gentamicin	0/11 (0%)
• Third generation cephalosporin	0/11 (0%)
***Staphylococcus aureus*** **(*****n*** **=** **9)**	
• Gentamicin	1/9 (11%)
• Oxacillin (methicillin-resistant -MRSA)	1/9 (11%)
**All early-onset sepsis isolates (*****n*** **=** **101)**	
• Benzylpenicillin + gentamicin (combined)	2/101 (2%)

*All group B streptococci (N = 60) and all viridans group streptococci (n = 10) were susceptible to benzylpenicillin*.

Among the nine *Staphylococcus aureus* EOS isolates, one isolates was nonsusceptible to both benzylpenicillin and aminoglycosides; the combination empiric regimen in use. Eight of nine isolates were susceptible to an aminoglycoside. One isolate was methicillin resistant (MRSA), but susceptible to an aminoglycoside. Six out of nine *S. aureus* EOS isolates were nonsusceptible to benzylpenicillin. All other Gram-positive EOS isolates in this study were uniformly susceptible to benzylpenicillin. The overall non-susceptibility rate to the current empirical regimen benzylpenicillin and gentamicin was 2/101 (2%) (Table 3).

## Discussion

This study reports the epidemiology of EOS and antibiotic susceptibility over more than two decades in a well-defined population in South-West Norway. Key findings are that GBS is the most common causative pathogen in EOS, but *E. coli* dominates in infants with GA <28 weeks. The incidence of EOS remained stable for the entire study period, but with a possible decline in incidence for the period 2000–2018 analyzed separately. Finally, we demonstrated a low level of antibiotic nonsusceptibility, and the current recommended empirical antibiotic regimen (benzylpenicillin and gentamicin) continues to have an excellent coverage for EOS in our area.

The overall incidence of EOS was 0.97/1000 LB, comparable to reports from other epidemiological studies from Sweden (0.9) (3), United Kingdom (0.7) (16) and the United States (0.77–1.08) (1, 5, 17). We found a 24-fold higher incidence of EOS in extremely preterm infants compared to term infants, a slightly lower ratio than the 30-fold higher EOS rate reported by Stoll et al. (5). The high rate of EOS cases in extremely preterm infants is a reminder of the vulnerability of preterm infants, emphasizing the importance of clinicians' high vigilance in care of these infants.

VGS, along with *E. coli*, was interestingly the second largest group of pathogens that caused EOS, predominantly in term infants. All infants with VGS-EOS had clinical symptoms suggestive of sepsis, CRP levels above 10 mg/L and received ≥5 days of antibiotics, indicating a clinically relevant sepsis episode. In many other studies, VGS are excluded as “unspecific” (3), complicating the comparisons between studies. Stoll et al. reported that 7/235 (3.0%) infants with EOS had growth of VGS (5), whereas 10/862 (1.2%) infants in the 1989–2003 data from Yale-New Haven had VGS sepsis (8). In the Yale-New Haven data, commensal organisms such as VGS increased during 1979–1988, possible because the simultaneously rise in preterm patient population with longer duration of NICU hospitalization and central vascular catheters. In our study though, VGS were almost exclusively found among term infants. VGS have also been reported to be the cause of EOS among infants exposed to chorioamnionitis and being asymptomatic within 6 h of age (18).

We found no change in the total incidence of EOS for the entire 23-year study period, nor in the incidence of Gram-positive or Gram-negative EOS. Data from Yale-New Haven Hospital showed a decrease in EOS from 1979 to 2004, and from 2004 to 2013 a stable incidence of around 0.9/1,000 (6). In contrast, a recent population-based study from Sweden showed a significant reduction in EOS incidence over the last two decades (3). However, both studies had a baseline incidence that were higher compared to our study. We found a significant reduction in the total EOS incidence with a mean 6% decline per year for the period 2000–2018. This was however not an a priori planned analysis, and should be interpreted with caution. On the other hand, it concurs with two multicenter studies from 2005–2014 in UK and 2002–2012 in Australian and New Zealand reporting a decreasing trend in the incidence of EOS to 0.7/1,000 and 0.83/1,000, respectively (16, 19).

*E. coli*, associated with high mortality rates, caused a substantial proportion of EOS in extremely preterm infants. We found a stable incidence of *E. coli* EOS during the 23 years, in line with other European studies (3, 16), but numbers may be too small to detect significant changes. In contrast, US and Australian studies (1, 5, 19–21) consistently reports higher rates of *E. coli* EOS. Stoll et al. reported an increasing incidence of *E. coli* sepsis when comparing surveillance data from 2015–2017 to 2006–2009 (5), with overall incidence rates of 0.4/1000 LBs and 12.1/1000 LB in infants with GA <28 weeks. The lower incidence of *E. coli* EOS in European studies are usually accompanied by a higher incidence rate of GBS, as in our study (3, 16). Yet, the incidence of GBS in our study is comparable to other reports (1, 5, 7). We did not find any change in incidence during these years. Risk-based intrapartum antibiotic preventive strategies against GBS, based on UK guidelines, were implemented in our unit in 2008 (22, 23). Limitations in the GBS prevention strategies have been demonstrated both in risk-based and in screening-based programs (5, 24), but the latter may be associated with a slightly lower rate of EOS caused by GBS.

We found that the maximum CRP level in both Gram-positive and Gram-negative bacteria were relatively low. This is in line with other studies reporting on elevation of inflammatory markers in EOS (25, 26), and is an important reminder for clinical evaluation of infants at risk of EOS within a structured strategy (4, 27).

One of the key findings in this study was that the vast majority (98%) of EOS isolates were susceptible to the current empirical antibiotic regimen; benzylpenicillin and gentamicin. Although numbers are small, the Gram-negative antibiotic nonsusceptibility rate, remained unchanged. This is comparable to a recent study, from the USA where they found a stable *E. coli* nonsusceptibility rate during 2009–2017 (10). The nonsusceptibility rates were on the other hand substantially higher than in our study. Our nonsusceptibility rates correlate well-with a UK study reporting a 7% nonsusceptibility to the empirical antibiotic regimen of benzylpenicillin and gentamicin among 328 EOS isolates (11). Furthermore, Cailes et al. reported a decreasing nonsusceptibility from 2005–2009 to 2010–2014, and also a lower nonsusceptibility rate among Gram-negative isolates causing EOS vs. late-onset sepsis. Nonsusceptibility rates are low in Norway due to a restrictive antibiotic policy, although increasing gentamicin nonsusceptibility of *E. coli*, extended-spectrum beta-lactamase producing Gram-negative bacteria and MRSA are increasingly found (28). This, correlates well-with Flannery et al. findings that pathogens causing EOS in the USA are not affected by the increasing extreme drug resistance seen globally (10, 29). In 2011, our NICU changed from ampicillin to benzylpenicillin as the beta-lactam backbone for empiric EOS therapy. Ampicillin use is associated with a higher *Klebsiella pneumoniae* gut colonization, including ampicillin nonsusceptible strains (30). Our results do not support the need for empirical use of ampicillin instead of benzylpenicillin.

Key strengths of this study are the 23-year long study period and the strictly population-based design. The number of infants included in our study roughly equals the total number of births in Norway over a 2-year period. We have complete data sets on all infants with positive blood cultures, including antibiotic susceptibility patterns, antibiotic use, and neonatal and maternal data for the entire study period. The data were collected by a single researcher. The study includes detailed maternal and neonatal information. We only included culture-confirmed episodes regarded as the “gold standard” for the definition of neonatal sepsis. There are also limitations. The data were collected from a single-center and the findings may not be generalizable to other countries with high antibiotic consumption driving high antibiotic nonsusceptibility rates, settings with higher burden of neonatal sepsis, or settings with limited resources and weak healthcare systems. Another important limitation is the low rate of extremely preterm infants. Albeit being in line with other regions of Norway, it may be lower compared to countries outside Scandinavia. Finally, there are inherent limitations with retrospective data.

## Conclusion

In this population-based study of EOS over 23 years, we found that that GBS was the most common causative pathogens in EOS, but among extremely preterm infants *E. coli* dominated. We found no change in the incidence of EOS during the whole 23-year period, but with a possible decrease in incidence during 2000–2018. Empirical benzylpenicillin and gentamicin in combination provides a very high coverage for EOS pathogens in our setting.

## Data Availability Statement

The raw data supporting the conclusions of this article will be made available by the authors, without undue reservation.

## Author Contributions

AV conceptualized and designed the project, collected and analyzed data, wrote the first version of the manuscript, and revised the manuscript. She had full access to all of the data in the study and takes responsibility for the integrity of the data and the accuracy of the data analysis. KØ conceptualized and designed the project, directed and organized all phases of the project, analyzed data, contributed with statistical analyses and supervised AV during all the phases of the project. CK and SR supervised AV during final phases of the project, analyzed data, revised the manuscript for intellectual content and approved the final manuscript. All authors approved the final manuscript as submitted and agree to be accountable for all aspects of the work.

## Conflict of Interest

The authors declare that the research was conducted in the absence of any commercial or financial relationships that could be construed as a potential conflict of interest.
